# The role for osmotic agents in children with acute encephalopathies: a systematic review

**DOI:** 10.1186/1471-2431-10-23

**Published:** 2010-04-17

**Authors:** Samson Gwer, Hellen Gatakaa, Leah Mwai, Richard Idro, Charles R Newton

**Affiliations:** 1 Centre for Geographic Medicine Research (Coast), KEMRI-Wellcome Trust Collaborative Research Programme, Kilifi, Kenya; 2 Joanna Briggs Evidence Synthesis Group, Kilifi, Kenya; 3 The International Centre of Insect Physiology and Ecology, Kenya; 4 Department of Paediatrics and Child Health, Mulago Hospital, Makerere University School of Medicine, Kampala, Uganda; 5 London School of Hygiene and Tropical Medicine, London, UK; 6 Department of Neurosciences, Institute of Child Health, University College London, UK

## Abstract

**Background:**

Raised intracranial pressure (ICP) is known to complicate both traumatic and non-traumatic encephalopathies. It impairs cerebral perfusion and may cause death due to global ischaemia and intracranial herniation. Osmotic agents are widely used to control ICP. In children, guidelines for their use are mainly guided by adult studies. We conducted this review to determine the current evidence of the effectiveness of osmotic agents and their effect on resolution of coma and outcome in children with acute encephalopathy.

**Methods:**

We searched several databases for published and unpublished studies in English and French languages, between January 1966 and March 2009. We considered studies on the use of osmotic agents in children aged between 0 and 16 years with acute encephalopathies. We examined reduction in intracranial pressure, time to resolution of coma, and occurrence of neurological sequelae and death.

**Results:**

We identified four randomized controlled trials, three prospective studies, two retrospective studies and one case report. Hypertonic saline (HS) achieved greater reduction in intracranial pressure (ICP) compared to mannitol and other fluids; normal saline or ringer's lactate. This effect was sustained for longer when it was given as continuous infusion. Boluses of glycerol and mannitol achieved transient reduction in ICP. Oral glycerol was associated with lower mortality and neurological sequelae when compared to placebo in children with acute bacterial meningitis. HS was associated with lower mortality when compared to mannitol in children with non-traumatic encephalopathies.

**Conclusion:**

HS appears to achieve a greater reduction in ICP than other osmotic agents. Oral glycerol seems to improve outcome among children with acute bacterial meningitis. A sustained reduction in ICP is desirable and could be achieved by modifying the modes and rates of administration of these osmotic agents, but these factors need further investigation.

## Background

Raised intracranial pressure (ICP) is a recognized feature of both traumatic and non-traumatic encephalopathies [1-4]. It impairs cerebral perfusion pressure (CPP), leading to ischaemia, and may cause death by compressing the brainstem during intracranial herniation. Raised ICP has consistently been shown to be an important determinant of outcome in children with central nervous system (CNS) infections and traumatic brain injuries (TBI)[1,4,5]. Management of raised ICP is aimed at optimizing CPP and oxygen supply to the brain in addition to reducing ICP. Methods to reduce ICP include postural changes, temperature regulation, hyperventilation, sedation, drainage of cerebro-spinal fluid, operative decompression, and the most widely used, osmotherapy [6-8].

Osmotherapy entails the use of pharmacologically inert substances that increase the osmotic pressure of plasma, promoting movement of water from interstitial space to vascular space[9]. Osmotic agents include mannitol, urea, sorbitol, glycerol and hypertonic saline (HS). Although these agents act mainly by reducing ICP via an osmotic gradient, they may have other beneficial effects. Thus, mannitol has been shown to scavenge reactive oxygen species[10], reduce the viscosity of blood, improving its flow through the circulation[11], and cause vasoconstriction, reducing cerebral blood volume[12]. Hypertonic saline is an effective volume expander which improves systemic haemodynamics and increases CPP[13]. In animal models, it has also been shown to enhance cerebral microcirculation by reducing adhesions of polymorphonuclear cells and by stimulating local release of nitric oxide[14].

Guidelines for use of osmotic agents have been developed from adult TBI studies. However, these have been adapted for children with minimal evidence obtained directly from children with TBI. Among children with non-traumatic encephalopathies, guidelines are virtually non-existent. In a postal survey of raised ICP management protocols in UK hospitals, practices varied greatly between hospitals[15]. Monitoring for patients with non-traumatic encephalopathy was seldom considered and thus, little consideration was given for use of osmotic agents in this group.

We performed this review to determine the best available evidence on the effectiveness of various osmotic agents and their effect on resolution of coma and outcome (neurological sequelae and mortality) in children with acute encephalopathies.

## Method

This review examines the effectiveness of osmotic agents in reducing ICP in children with acute encephalopathies and, the effect of osmotic agents on resolution of coma and clinical outcome (neurological sequelae and mortality) in children with acute encephalopathies.

### Inclusion criteria

We searched published and unpublished studies in the English and French languages between January 1966 and March 2009. We reviewed randomized controlled trials with the aim of performing a meta-analysis. In addition, we examined quasi- and non-randomized clinical trials, case control, cohort, and before and after studies, case series, and case reports, for consideration in a narrative summary.

We evaluated studies that included children aged between 0 and 16 years with acute traumatic and non-traumatic encephalopathies, characterized by altered consciousness. Agents included in our search were mannitol, hypertonic saline, urea, sorbitol and glycerol. The primary outcome measure was reduction in ICP. Secondary outcome measures were resolution of coma and clinical outcome (neurological sequelae and death).

### Search Strategy

We searched Pubmed, Cochrane library, EMBASE and cumulative index to nursing and allied health literature (CINAHL). Other databases included were current controlled trials, the trials register for promoting health interventions (TRoPHI), Australian clinical trials registry (ACTR), clinical medicine net prints collection, Bandolier evidence based health care, and the center for clinical trials and evidence-based healthcare at Brown medical school. The search databases for unpublished studies and grey literature were dissertation abstracts international, the World Health Organization library, Agency for Healthcare Research and Quality, Grey literature report, National Library of Medicine, theses Canada portal, Proquest digital theses, Australasian digital theses program and the British library. The initial search analyzed the text words contained in the title and abstract, and the index terms used to describe the articles. A second search that used all identified keywords and index terms, individually and in combinations, was applied. The reference list of all identified reports and articles were then searched for additional studies. In our search in Pubmed, we applied the search phrase "(mannitol [MeSH] OR hypertonic saline [MeSH] OR urea [MeSH] OR sorbitol [MeSH] OR glycerol [MeSH]) AND (hepatic encephalopathy [MeSH] OR malaria, cerebral [MeSH] OR meningitis [MeSH] OR encephalitis [MeSH] OR brain injuries [MeSH] OR head injuries [MeSH] OR coma [MeSH] OR intracranial hypertension [MeSH])" and limited it to children, the English and French languages, and the duration between 1^st ^of January 1966 and 31^st ^March 2009, and children."

### Assessment of studies

The papers selected for retrieval were assessed by two independent reviewers for methodological validity prior to inclusion in the review. We used the standardized critical appraisal instruments from the Joanna Briggs Institute Meta-Analysis of Statistics Assessment and Review Instrument (JBI-MAStARI) Critical Appraisal tool[16]. Any disagreements between the reviewers were resolved through discussion, or with a third reviewer. We extracted data using the standardized JBI data extraction tool[16]. All data were entered twice and discrepancies resolved. We considered quantitative studies for pooling for statistical meta-analysis. Relative risk and their 95% confidence intervals (95%CI) were calculated for analysis. Other findings were presented in a narrative form.

The protocol that guided this review is available, on request, from the review protocols section of the Joanna Briggs institute website; http://www.joannabriggs.edu.au/pubs/systematic_reviews_prot.php

## Results

**Figure 1:**
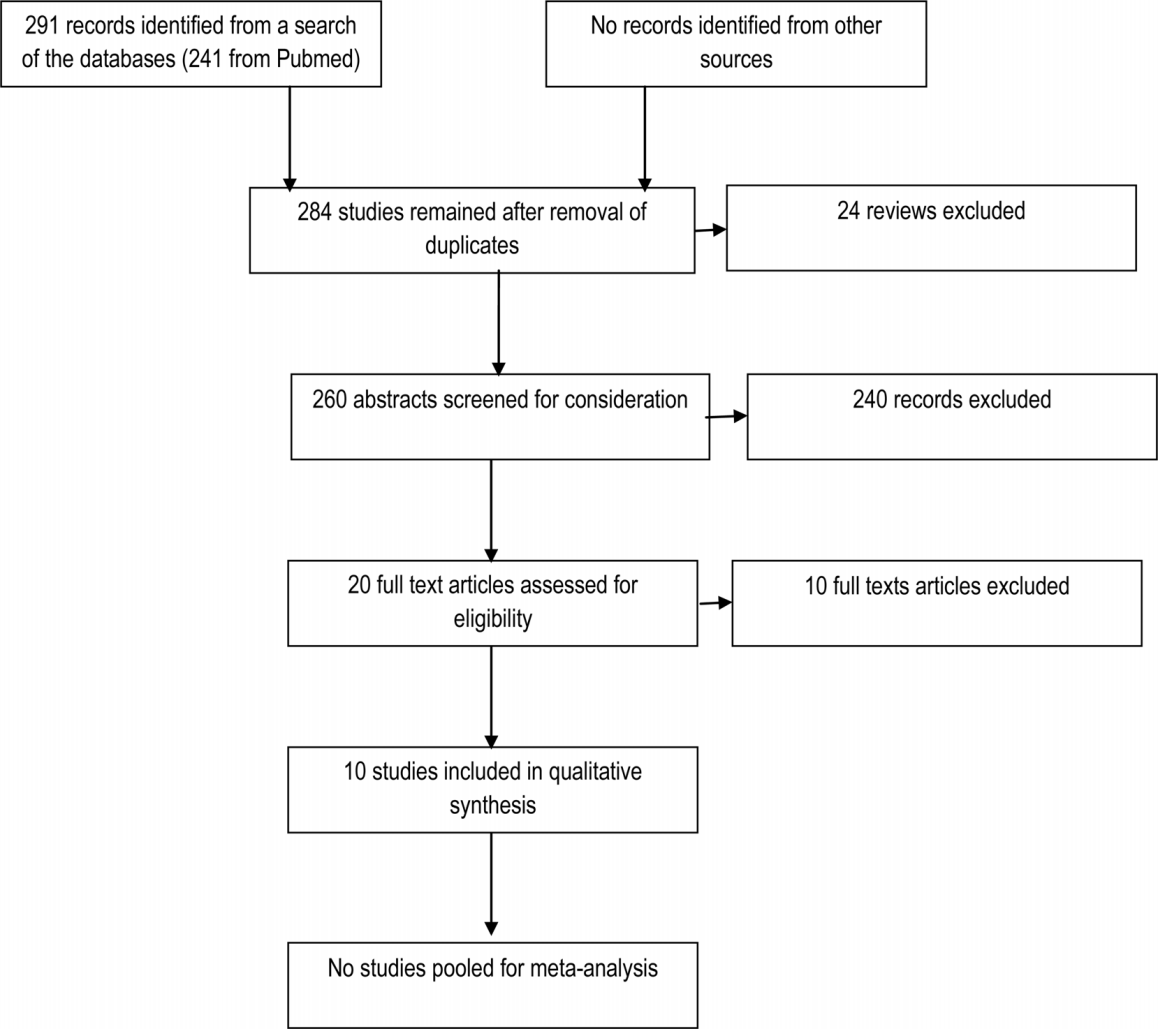
**Flow of information through the systematic review**.

Our search of the databases revealed 291 records. We identified 20 studies that met our review criteria. We critically appraised them using the JBI-MASTARI assessment tool and excluded 10 studies. The different phases of the review are summarized in figure 1. Four of the studies excluded did not provide any data or information on the relationship between the interventions and the outcomes of interest [17-20]. Three studies included children and adults but data on children were not provided separately nor could we obtain this information from the authors [21-23]. Two other studies were excluded for other reasons[24,25] (Table 1).

**Table 1:** Reason for exclusion of studies

Study	Reason for exclusion
Prabhakaran 2004[19]	The paper did not report on effect of the intervention, mannitol, on ICP or outcome
Vialet 2003[23]	The study provides combined results for adults and children, and the data for children could not be extracted
Kingston 1971[18]	There is little information on the characteristics of the participants. There is no data provided on the effect of the intervention, urea, on ICP or outcome
Cruz 2002[17]	The use of the intervention, mannitol, was not clearly evaluated and the study does not demonstrate the relationship between mannitol use and outcome
James 1977[22]	The study provides combined the results for adults and children, and the data on children could not be extracted.
James 1980[21]	The study provides combined the results for adults and children, and the data on children could not be extracted.
MacDonald 1982[25]	Reason for selective data presentation not given and administration of both treatments not clearly described
Marshall 1978[24]	Age of subjects is not given and unclear statistical methods have been used
Mickell 1977[20]	The relationship between the intervention and ICP or outcome is not described
Procaccio 1991[36]	The study included a heterogeneous age group of patients that is not comparable and in whom standard treatment was not provided to all

We included four randomized controlled trials (RCTs) [26-29], one of which was a cross-over trial[26], three prospective observational studies[1,30,31], two retrospective studies[32,33], and one case report[34]. Out of these, four studies involved patients with non-traumatic encephalopathies [1,27,28,32]. The characteristics of these studies (participants, interventions, comparisons, outcomes, and study design) are summarized in additional file 1. Among the clinical trials, assignment of treatment was random in all the studies, but in two studies, the allocation to treatment groups was not concealed from the investigators [26,29]. We assessed each study according to its study design as shown in tables 2, 3 and 4.

**Table 2:** Methodological assessment of clinical trials

Study	Fisher **1992 **[26]	Namutangula **2007 **[27]	Peltola **2007 **[28]	Simma **1998 **[29]
Was the assignment to treatment groups truly random?	Y	Y	Y	Y
Were participants blinded to treatment allocation?	N	Y	Y	Y
Was allocation to treatment groups concealed from the allocator?	U	Y	Y	N
Were the outcomes of people who withdrew described and included in the analysis?	Y	Y	Y	Y
Were those assessing outcomes blind to the treatment allocation?	U	Y	Y	N
Were the control and treatment groups comparable at entry?	N	Y	Y	Y
Were the groups treated identically other than for the named interventions?	Y	Y	Y	Y
Were outcomes measured in the same way for all groups?	Y	Y	Y	Y
Were outcomes measured in a reliable way?	Y	Y	Y	Y
Was appropriate statistical analysis used?	Y	Y	Y	Y

* Y = Yes, N = No, U = Unclear

**Table 3:** Methodological assessment of cohort study

Study	Yildizdas **2006 **[32]
Is sample representative of patients in the populations as a whole?	Y
Are the patients at a similar point in the course of their condition/illness?	Y
Has bias been minimized in relation to selection of cases and of controls?	U
Are confounding factors identified and strategies to deal with them stated?	N
Are outcomes assessed using objective criteria?	Y
Was follow up carried out over a sufficient time period?	Y
Were the outcomes of people who withdrew described and included in the analysis?	Y
Were outcomes measured in a reliable way?	Y
Was appropriate statistical analysis used?	Y

* Y = Yes, N = No, U = Unclear

**Table 4:** Methodological Assessment of descriptive and case series studies

Study	**Berger 2002 **[34]	Peterson **2000 **[33]	Khanna **2000 **[30]	Newton **1997 **[1]	Wald **1982 **[31]
Was study based on a random or pseudo-random sample?	N	N	N	N	N
Were the criteria for inclusion in the sample clearly defined?	Y	Y	Y	Y	N
Were confounding factors identified and strategies to deal with them stated?	N	N	U	U	U
Were outcomes assessed using objective criteria?	Y	Y	Y	Y	Y
If comparisons were being made, were there sufficient descriptions of the groups?	Y	Y	Y	Y	Y
Was follow up carried out over a sufficient time period?	Y	Y	Y	Y	Y
Were the outcomes of people who withdrew described and included in the analysis?	Y	Y	Y	Y	Y
Were outcomes measures in a reliable way?	Y	Y	Y	Y	Y
Was appropriate statistical analysis used?	Y	Y	Y	Y	Y

* Y = Yes, N = No, U = Unclear

### Intracranial Pressure

ICP was monitored in 7 studies; 2 RCTs[26,29], 4 observational studies[1,30,31,33] and one case report[34]. In one RCT, ringer's lactate was compared to HS for resuscitation of 32 children with traumatic brain injuries[29]. More interventions for raised ICP were used in the Ringer's lactate group compared to the HS group (P < 0.01)[29]. However, there was no significant difference in the mean ICPs between the two groups after the interventions. In a crossover trial that included 18 children with TBI, there was a significant drop from the initial ICP with use of HS(P = 0.003) compared to normal saline (P = 0.32)[26].

Hypertonic saline given as a continuous infusion in a study of 10 children with TBI achieved a significant and sustained reduction of ICP that was maintained over 72 hours (P < 0.01)[30]. These children had raised ICP that was refractory to other management strategies including the use of bolus infusions of mannitol. Among 23 children with cerebral malaria, a dose-response effect with use of boluses of mannitol was observed in moderately raised ICP (ICP > 20 mmHg, CPP < 50 mmHg) but not with severely raised ICP (ICP > 40 mmHg, CPP < 40 mmHg)[1]. In a study of 3 children with TBI, oral glycerol was shown to reduce ICP by at least 50% within the first half hour of administration and maximally after 60 minutes[31]. This reduction was not maintained beyond 90 minutes. A case report of two children with TBI showed a dose response relationship between both HS and mannitol, and ICP [34]. However, mannitol appeared to cause a reduction in CPP.

All the five studies that investigated HS[26,29,30,33,34] demonstrated a dose-response effect on ICP irrespective of the saline concentrations.

### Mortality

**Figure 2:**
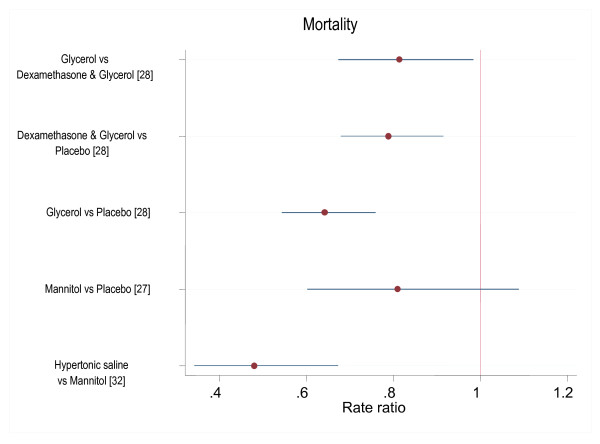
**Risk ratios of death with different osmotic agents**.

All the included studies reported mortality. However, in examining mortality, we only analyzed the RCTs since they were the only studies that had groups for comparison. The four RCTs [26-29] identified were heterogeneous in relation to the interventions used and could not be pooled for meta-analysis. In one multicentre trial on 654 children with bacterial meningitis, the mortality was lower in those given glycerol compared to placebo (RR 0.64 95%CI 0.54, 0.76)[28] and, in children given glycerol and dexamethasone combination compared to placebo (RR 0.79 95%CI 0.68, 0.92)[28] (Figure 2). Our own analysis of this data suggests a lower mortality with use of glycerol and dexamethasone combination compared to glycerol alone (RR 0.81 95% CI 0.67, 0.98). Another trial comparing the use of HS and ringer's lactate as resuscitative fluids in 32 children with TBI reported only 2 deaths which occurred in children receiving ringer's lactate[29]. Among 156 children with cerebral malaria (CM), there was no observed difference in mortality when a single bolus of mannitol was administered compared to normal saline (RR 0.81 95%CI 0.60, 1.09)[27]; this trial was however not powered to detect a difference in mortality. In a clinical series of children with non-traumatic encephalopathies, there was less mortality with use of HS compared to mannitol (RR 0.48 95%CI 0.34, 0.67)[32].

### Neurological sequelae

Four studies reported on neurological sequelae[1,28,30,33], but only one of these studies was a clinical trial[28]. In this trial examining the use of glycerol and dexamethasone in 654 children with bacterial meningitis, poor outcome (defined as severe neurological sequelae and profound hearing loss) was lower in the glycerol group (RR 0.58 95%CI 0.50, 0.67) and the glycerol and dexamethasone combination (RR 0.55 95%CI 0.47, 0.65) compared to placebo [28]. This lower risk was similarly observed when severe neurological sequelae were examined alone. No significant differences were observed between the glycerol and the glycerol and dexamethasone combination.

### Resolution of Coma

Only one study, a clinical trial of mannitol on children with CM, examined resolution of coma as an outcome measure[27]. There was no difference between use of mannitol and placebo in coma resolution [median (IQR duration 20.5 (14.1-53.4) and 18.9 (10.0-38.0) hours respectively (p = 0.11)].

## Discussion

We identified four RCTs, one of which was on children with non-traumatic encephalopathies. Each trial compared different agents and could not be pooled for meta-analysis. We examined 3 prospective observational studies, 2 retrospective studies and 1 case report. In our evaluation, HS appeared to achieve greater reduction in ICP than other osmotic agents. Oral glycerol was associated with less mortality and neurological sequelae when compared to placebo among children with acute bacterial meningitis. The only study that examined resolution of coma did not show any difference between the osmotic agent, mannitol, and placebo.

There was a dose response in the reduction of ICP with use of all the agents examined. However, ICP is a dynamic entity and single measurements on admission indicating raised ICP do not predict clinical outcome[1]. The analysis of ICP measurements therefore often consists of determining the duration of time that ICP is above a certain threshold[35]. It is desirable that interventions result in sustained reductions in ICP. And so, whilst all agents in the studies reviewed exhibited a dose response effect, this was transient in a number of cases. Continuous infusions of HS appeared to achieve sustained reduction in ICP. However, the advantage of different rates of administration can only be reliably investigated in clinical trials that specifically investigate the different modes and rates of administration of a particular intervention.

Hypertonic saline was shown to reduce ICP more than either normal saline [26] or ringer's lactate solutions[29]. When compared to mannitol, HS maintained or improved CPP, an important determinant of neurological outcome[34]. This effect is particularly important in acute encephalopathies associated with volume deficits such as TBI and cerebral malaria. Theoretically, HS as a crystalloid may equilibrate freely throughout brain tissue in encephalopathies associated with impairment of the blood brain barrier such as meningitis, thus aggravating ICP. However there is little evidence of this phenomenon in the studies that we reviewed.

There was a lower relative risk of death with HS compared to mannitol[32], and with oral glycerol or a combination of oral glycerol and dexamethasone compared to placebo[28]. In the latter trial, glycerol, either alone or combined with dexamethasone, was associated with fewer neurological sequelae compared to placebo. There appeared to be less mortality when glycerol was used in combination with dexamethasone compared to glycerol used alone. Glycerol, given orally, allows for a convenient mode of administration considering resource poor settings. This trial was carried out across 10 institutions in different countries and the consistency of care is likely to have been difficult to maintain. Studies needed to examine effects of osmotic agents on neurological outcome would require large samples sizes and longer durations of follow up than those of the studies we examined.

We restricted our search to studies that were published in the English and French languages after 1966, potentially missing out on a number of studies in other languages. However, it is unlikely that a search of literature before 1966 could have yielded adequately reported studies on children. Most of the studies that we have included are observational studies and thus limit the validity of our analysis.

In one study that we included[29], the osmotic agent was examined for use as a resuscitative fluid, not for treatment of ICP. Nevertheless, this study provided data on the use of osmotic agents and suggested a more effective mode of administration of osmotic agents, supporting continuous rather than bolus infusions. We have included one study that had a mixed population of children and adults[31]. In this study, some of the data on children is provided separately. In another study, young infants were also recruited but their results were not analysed separately[32]. Young infants have an immature nervous system and a patent anterior fontanelle, and the dynamics of ICP is different from that of older children. In addition, scoring them for coma using a similar scale as for older children could be misleading. Another study investigated children with a varied aetiology of acute encephalopathies[36]. Even so, the pathophysiology of raised ICP appears to be similar irrespective of aetiology.

A clinical trial examining the use of oral glycerol and rectal paracetamol among children with meningitis in Malawi (ISRCTN70121840) is ongoing and when complete, may provide more insight on the use of osmotic agents in children.

## Conclusion

The review supports the use of oral glycerol in children with acute bacterial meningitis and the use of hypertonic saline in acute traumatic and non-traumatic encephalopathies. However, the evidence presented is not sufficient to provide guidelines. Further clinical trials are needed to examine the safest and most efficacious concentrations of the various agents, particularly hypertonic saline. Such studies will also guide on the appropriate routes of administrations and the optimum rates of administration of these agents. Multi-centre trials may be necessary to achieve adequate sample sizes.

## Competing interests

The authors declare that they have no competing interests.

## Authors' contributions

SG conceived the review. SG, HG and LM researched for the review. HG provided statistical support. All the authors participated in drafting the manuscript. All the authors have read the approved final version.

## Authors' information

SG, HG and LM are members of the Joanna Briggs Institute (JBI) evidence synthesis group (Kenya chapter) and were supported by the JBI program. CN is supported by the Wellcome Trust, UK (070114).

## Pre-publication history

The pre-publication history for this paper can be accessed here:

http://www.biomedcentral.com/1471-2431/10/23/prepub

## Supplementary Material

Additional file 1 **Characteristics of included studies**. This table provides a summary of the characteristics of included studies, including details regarding participants, interventions, comparison groups, outcome, and the study design. The authors' conclusions and the reviewers' comments on each paper are also included Click here for file
