# Supplementary material for: The role for osmotic agents in children with acute encephalopathies: a systematic review
Source: BMC Pediatr. 2010 Apr 17;10:23. doi: 10.1186/1471-2431-10-23 (PMC2859077; doi:10.1186/1471-2431-10-23)
Supplement: Additional file 1 — Characteristics of included studies. This table provides a summary of the characteristics of included studies, including details regarding participants, interventions, comparison groups, outcome, and the study design. The authors' conclusions and the reviewers' comments on each paper are also included [file 1471-2431-10-23-S1.DOC]

**Characteristics** of included studies

| **Study** | **Study design, setting, participants** | **Interventions/**  **comparison groups** | **Outcome** | **Author’s conclusions** | **Reviewer’s comments** |
| --- | --- | --- | --- | --- | --- |
| Peltola 2007[28] | Design:  Multicentre RCT  Setting:  Multiple health institutions, South America  Participants:  Children with ABM  N=654 | A. Oral glycerol 1.5g/Kg QID  (n=166)  B. Oral glycerol 1.5g/Kg & Dexamethasone 0.15mg/Kg  QID  (n=159)  C. Placebo  (n=163)  D. Dexamethasone  (n=166) | *Death*  A = 17 B = 20 C = 26 D= 23 (p = 0.383, determined by the x^2^ tests between the 4 groups)  *Severe Neurological sequelae*  A = 7 (P = 0.01) B = 8 (P = 0.03) C = 19 D= 10 (p = 0.022, determined by the x^2^ tests between the 4 groups)  *Profound hearing loss*  A = 12 B = 9 C = 12 4D= 10 (p = 0.879, determined by the x^2^ tests between the 4 groups) | Neurological sequelae alone, and combined death and neurological sequelae, occurred with significantly less frequency in the Glycerol (A) and Glycerol and Dexamethasone groups (B) compared to the placebo (C) and Dexamethasone groups. (D). Hearing loss occurred with similar frequency in the 4 groups.  The incidence of severe neurological sequelae was significantly lower in all the treatment groups compared to the placebo. | The study is comprehensive and well designed to measure the effects of the agents. It demonstrates an obvious advantage in the use of glycerol alone or glycerol with dexamethasone, over placebo, in reducing severe neurological sequelae. However, the assessment of neurological sequelae at discharge was not sufficiently comprehensive to evaluate overall neurological outcome and the assessment should ideally have been repeated a few months after discharge. Withdrawals are described but are not included in per-protocol analysis. There were some protocol differences between the different sites. Dexamethasone use has not been examined in our review. |
| Namutangula 2007[27] | Design: RCT  Setting: Children’s emergency ward, Mulago hospital, Uganda  Participants: Children with CM  N=156 | Mannitol 1g/Kg  (n = 76)  Placebo  (n = 80) | *Death*  Mannitol = 10 Placebo = 13 (RR = 1.2, C.I. 0.5, 2.7)  *Median time to regain consciousness*  Mannitol = 18.9 hrs Placebo = 20.5 hrs (P = 0.11) | Mannitol does not significantly reduce time taken to regain consciousness or mortality. | The sample size was too small to determine effect of mannitol on mortality.  ICP is not measured and as such, it is not determined if all the patients warranted treatment with mannitol. Thus the potential effect of mannitol may not be detected.  Mannitol is administered as an initial single dose. ICP is dynamic and mannitol has a limited duration of action. The single dose is unlikely to have been adequate. |
| Simma 1998[29] | Design: RCT  Setting: ICU, Zurich children’s hospital, Switzerland  Participants: Children with TBI N=32 | Hypertonic saline  (HS) (n = 15)  Ringer’s Lactate  (RL) (n = 17) | Death  HS = 0 RL = 2  Greater need for other interventions to keep ICP at ≤ 15mmHg in RL patients compared to the HS patients  (p < 0.01) | Use of hypertonic saline for resuscitation and fluid management during the first 3 days after severe head injury is associated with lower ICP, higher cerebral perfusion and fewer adverse events compared to Ringer's Lactate. Children who received Hypertonic saline remained comatose for shorter durations and had less mortality | In this study, hypertonic saline is being examined as a resuscitative fluid rather than a direct intervention for raised ICP. In cases where ICP was raised, the patients received specific therapy which in some cases included Mannitol. Even so, they are still able to demonstrate lower incidence of raised ICP and death in children who received hypertonic saline compared to those who received ringers lactate. |
| Fisher 1992[26] | Design: Double blind, cross-over trial  Setting: Paediatric trauma service, children’s hospital of San Diego, USA  Participants: Children with TBI  N=18 | 3% HS  0.9% Saline  (Crossover study) | *Change in ICP*  3% HS: Initial ICP = 19.9mmHg  Average ICP = 15.8mmHg (P = 0.003)  0.9% Saline:  Initial ICP = 19.3 mmHg  Average ICP = 20.0 mmHg  (P = 0.32) | Acute hypernatraemia achieved with use of 3% HS is associated with decreased ICP in pediatric patients over a short period. | There is an evident dose response effect with use of hypertonic saline (3%) but the duration of observation is too short to examine for sustained response. A similar effect on ICP is not seen with 0.9% saline and in 10 instances, the ICP rose warranting other interventions. Standard deviations are not provided for the means of ICP after interventions of either agent.. However, 3% saline appears to be more effective than 0.9% saline in managing raised ICP. |
| Khanna 2000[**30]** | Design: Prospective observational study  Setting: Paediatric ICU, San-Diego Children’s hospital, USA  Participants: Children with TBI  N=10 | 3% HS infusion | *Change in ICP*  There was decrease in ICP between time 0 and at 6, 12, 24, 48 and 72 hours after initiation of therapy (p < 0.01)  There was a decrease in ICP spike frequency at 6, 12 ,24,48 and 72 hours after start of therapy (p < 0.01) | Hypertonic saline effectively controls intracranial hypertension resistant to conventional therapy. There is a statistically significant relationship between serum sodium and ICP. The clinical outcome in these patients is impressive as a result of hypertonic saline use. Only one child died and the authors attribute this to late presentation. | There is an apparent dose-response relationship between use of hypertonic saline and ICP. However, lack of a comparison group restricts conclusion on effect on clinical outcome. |
| Newton 1997[1] | Design: Prospective observational study  Setting: Children’s HDU, Kilifi district hospital, Kenya  Participants: Children with CM and moderate or severe ICP  N=13 | Mannitol  0.5-1g/Kg | *Change in ICP*  Mannitol reduced ICP in all instances. | Mannitol was effective in reducing ICP in children with moderate ICP but not in those with severe ICP. This change was not sustained in a number of occasions and was not examined systematically for significance. It was not clear if mannitol influenced outcome. | An infusion of mannitol at all times resulted in a reduction in ICP but this was not sustained in several instances and among those with severe ICP, did not even achieve intended therapeutic target on several occasions of use. Because of the design of this study, it is difficult to relate the use of mannitol to outcome. |
| Wald 1982[31] | Design: Prospective observational study  Setting: Department of neurosurgery, university of Cincinnati medical centre  Participants: Children with TBI and ICP>300mm of H_2_O  N=3 | Glycerol 0.5-1g/Kg every 3-4 hrs | *Change in ICP*  Mean ICP reduced in the children after glycerol infusion with lowest ICP being achieved at 60 minutes after which it appeared to gradually rise | Glycerol use resulted in reduction of ICP independent of initial level of pressure. | Examination of other outcomes cannot be done as all patients (adults and children) are grouped together and data specific to children is not provided. |
| Yildizdas 2006[32] | Design: Retrospective study  Setting: PICU, Cukurova university school of medicine, India  Participants: Children with non-traumatic encephalopathies  N=67 | I -Mannitol 0.5g/Kg  (n = 22)  II – HS  (n = 25)  III - Mannitol 0.5g/Kg and HS  (n = 20) | *Death*s  I = 11 II = 6 III = 3 (P = 0.003)  *Resolution of coma (mean)*  I = 123 hrs II = 88.6 hrs III = 87.5 hrs (P = 0.004) | The administration of HS is safer and more effective than mannitol. Groups II (HS only) and III (HS and Mannitol) showed better results in relation to duration of coma and mortality.  The authors acknowledge the lack of ICP measurement as a disadvantage in their study and conclude that proper timing of treatment requires ICP monitoring. | Combining HS and Mannitol as an intervention was not very informative since ICP monitoring was not done. The combined group was categorized into 2; those who received both agents (A) and those who received hypertonic saline after stopping mannitol (B). The outcomes were not similarly grouped. For the purpose of our review, we have excluded this group.  It is not clear whether treatment allocation was done randomly or was period specific.  The study includes young infants, a group that needs to be analysed separately because they have immature nervous system and patent fontanelle. This also has a bearing on scoring for coma which is one of the outcomes examined. |
| Peterson 2000[33] | Design : Retrospective  Study  Setting: USA  Participants: Children with traumatic brain injury and ICP>20mmHg  N=68 | 3% HS infusion | *Change in ICP*  No quantitative data is provided on this | Continuous infusion of HS was efficacious and safe for use in managing raised ICP in paediatric TBI. | ICP monitoring was done but quantitative data to show a dose response effect is not provided. Outcome is well examined for but the lack of a comparison group makes it difficult to conclude on effect of HS use on clinical outcome. |
| Berger 2002[34] | Case report  Setting: Paediatric ICU, Johannes Gutenberg university, Germany  Participants: 11 and 12 year old children with traumatic brain injury | 20% HS  and  20% Mannitol | *Change in ICP*  Bolus administration of either agents resulted in reduction in ICP. This reduction was not sustained in a number of occasions. | HS appeared to reduce post-traumatic increased ICP and improve CPP more effectively than comparable amounts of mannitol. | A dose response effect on ICP with use of both osmotic agents is demonstrated but mannitol appears to cause a reduction in CPP. |
